# Elevated insulin-like growth factor 1 receptor signaling induces antiestrogen resistance through the MAPK/ERK and PI3K/Akt signaling routes

**DOI:** 10.1186/bcr2883

**Published:** 2011-05-19

**Authors:** Yinghui Zhang, Marja Moerkens, Sreenivasa Ramaiahgari, Hans de Bont, Leo Price, John Meerman, Bob van de Water

**Affiliations:** 1 Division of Toxicology, Leiden/Amsterdam Center for Drug Research, Leiden University, Einsteinweg 55, NL-2333 CC, Leiden, The Netherlands

## Abstract

**Introduction:**

Insulin-like growth factor 1 (IGF-1) receptor (IGF-1R) is phosphorylated in all breast cancer subtypes. Past findings have shown that IGF-1R mediates antiestrogen resistance through cross-talk with estrogen receptor (ER) signaling and via its action upstream of the epidermal growth factor receptor and human epidermal growth factor receptor 2. Yet, the direct role of IGF-1R signaling itself in antiestrogen resistance remains obscure. In the present study, we sought to elucidate whether antiestrogen resistance is induced directly by IGF-1R signaling in response to its ligand IGF-1 stimulation.

**Methods:**

A breast cancer cell line ectopically expressing human wild-type IGF-1R, MCF7/IGF-1R, was established by retroviral transduction and colony selection. Cellular antiestrogen sensitivity was evaluated under estrogen-depleted two-dimensional (2D) and 3D culture conditions. Functional activities of the key IGF-1R signaling components in antiestrogen resistance were assessed by specific kinase inhibitor compounds and small interfering RNA.

**Results:**

Ectopic expression of IGF-1R in ER-positive MCF7 human breast cancer cells enhanced IGF-1R tyrosine kinase signaling in response to IGF-1 ligand stimulation. The elevated IGF-1R signaling rendered MCF7/IGF-1R cells highly resistant to the antiestrogens tamoxifen and fulvestrant. This antiestrogen-resistant phenotype involved mitogen-activated protein kinase/extracellular signal-regulated kinase (MAPK/ERK) and phosphatidylinositol 3-kinase/protein kinase B pathways downstream of the IGF-1R signaling hub and was independent of ER signaling. Intriguingly, a MAPK/ERK-dependent agonistic behavior of tamoxifen at low doses was triggered in the presence of IGF-1, showing a mild promitogenic effect and increasing ER transcriptional activity.

**Conclusions:**

Our data provide evidence that the IGF-1/IGF-1R signaling axis may play a causal role in antiestrogen resistance of breast cancer cells, despite continuous suppression of ER transcriptional function by antiestrogens.

## Introduction

Acquisition of antiestrogen resistance is a common impediment in endocrine therapy for estrogen receptor (ER)-positive breast cancer. It is therefore imperative to understand the underlying mechanisms of resistance to identify novel therapeutic targets for treatment of resistant breast cancers.

The molecular mechanisms of antiestrogen resistance are intricate. The canonical ER pathway responds to estrogen to initiate a series of cell growth events via ER cofactors, cell cycle regulators, cell survival and apoptosis mediators [1-4]. Compelling evidence from clinical and experimental settings links antiestrogen resistance to elevated signaling of receptor tyrosine kinases (RTKs) such as the members of the epidermal growth factor receptor (EGFR) family, EGFR and human epidermal growth factor receptor 2 (HER2) [1-4]. Altered expression and activation of EGFR/HER2 and their key downstream signaling components, mitogen-activated protein kinase/extracellular signal-regulated kinase (MAPK/ERK) and phosphatidylinositol 3-kinase/protein kinase B (PI3K/Akt), can elicit antiestrogen resistance, either through phosphorylation of ER, such as at Ser167 by EGFR/Akt and at Ser118 by HER2/ERK to increase ER DNA binding and ER coactivator interaction, or via other independent pathways, such as upregulated antiapoptotic machinery of B-cell lymphoma 2 (Bcl-2) and B-cell lymphoma extra large (Bcl-xL) [1,4-7]. Furthermore, high EGFR/HER2 RTK signaling not only may promote *de novo *and acquired antiestrogen resistance but also may signal in an ER-independent manner, thereby promoting cell proliferation in its own right [1,4-7]. Signaling networks assembled by RTKs are therefore critical contributors to the development of breast cancer resistance to antiestrogens.

In addition to EGFR/HER2, there is increasing evidence for the involvement of the insulin-like growth factor 1 (IGF-1) receptor (IGF-1R) in antiestrogen resistance. IGF-1R, as part of the large class of RTKs, is now considered a potential cellular oncogene that plays a key role in various cellular processes, such as proliferation, survival, transformation, differentiation as well as cell-cell and cell-substrate interactions [8,9]. In breast neoplastic cell lines, expression of IGF-1R is a fundamental prerequisite for a malignant phenotype, potentially facilitating cell survival and metastasis [8,10-12]. Clinically, IGF-1R is commonly overexpressed in primary breast tumors [13,14] and phosphorylated in all breast cancer subtypes, correlating with poor survival [15]. In ER-positive breast cancer cells, IGF-1R and ERα are often coexpressed and respond to the synergistic action of estrogen and IGF-1 signaling, leading to cross-talk between the ER and IGF-1R pathways [16,17]. In tamoxifen-resistant breast cancer cells, IGF-1R is upregulated [18-20] and acts upstream of estrogen-activated EGFR [21,22]. Moreover, IGF-1R confers resistance by forming a heterodimer with HER2, allowing HER2 signaling to resume in the presence of trastuzumab [23]. While the accumulating data just described show that IGF-1R operates through signaling cross-talk with estrogen-ER signaling and EGFR/HER2 regulatory pathways in antiestrogen-resistant breast cancer cells, the ER-independent role of IGF-1R signaling in antiestrogen resistance is poorly understood [1,4].

To elucidate the direct role of IGF-1R signaling in breast cancer antiestrogen resistance, we established an ER-positive human breast cancer cell line ectopically expressing human wild-type IGF-1R, MCF7/IGF-1R, with elevated IGF-1R tyrosine kinase activity. In the present study, we demonstrate that while MCF7/IGF-1R cells remain antiestrogen-responsive, IGF-1 ligand stimulation induces rapid and sustained IGF-1R/MAPK/PI3K signaling and directly causes an ERα-independent resistance to the antiestrogens tamoxifen and fulvestrant (FUL) in two-dimensional (2D) as well as 3D culture. In addition, tamoxifen at low doses functions as an agonist in IGF-1-stimulated MCF7/IGF-1R cells, further increasing IGF-1-dependent proliferation. Our results indicate that IGF-1R signaling can be a single determinant for antiestrogen efficacy and hence suggest that IGF-1R and the key components involved in the IGF-1R signaling network are potential targets in combined antiestrogen therapy.

## Materials and methods

### Antibodies and reagents

Antibodies to rabbit anti-phospho-IGF-1Rβ (Tyr1131), anti-phospho-IGF-1Rβ (Tyr1135/Tyr1136), anti-ERK1/2, anti-phospho-ERK1/2 (Thr202/Tyr204), anti-Akt and anti-phospho-Akt (Ser473) (Cell Signaling Technology Danvers, MA, USA), mouse anti-IGF-1Rβ and rabbit anti-ERα (Santa Cruz Biotechnology, Santa Cruz, CA, USA), and mouse antitubulin (Sigma-Aldrich, St. Louis, MO, USA) were commercially purchased. Conjugated secondary antibodies included Alexa Fluor 488 antimouse (Jackson ImmunoResearch, West Grove, PA, USA), antimouse horseradish peroxidase (HRP) and antirabbit HRP (Jackson ImmunoResearch, West Grove, PA, USA), and antirabbit alkaline phosphatase (AP) (Tropix Western-SuperStar™ Immunodetection System; Applied Biosystems, Foster, CA, USA). Human IGF-1 (Sigma-Aldrich) was prepared in sterile H_2_O (100 μg/mL). The estrogen compound 17β-estradiol (E2) and the antiestrogens 4-hydroxytamoxifen (4-OH-TAM) and FUL (Sigma-Aldrich) were dissolved in dimethyl sulfoxide (DMSO) to 1 mM stock concentration. The IGF-1R inhibitor BMS-536924 and dual PI3K/mammalian target of rapamycin (mTOR) inhibitor BEZ235 (Selleck Chemicals LLC Houston, TX, USA) and the mitogen-activated protein kinase kinase (MEK) inhibitor U0126 (Promega, Madison, WI, USA), were prepared in DMSO at 10 mM stock concentration.

### Cell culture, retrovirus production and establishment of IGF-1R stably overexpressing human breast cancer cell line

MCF7 cells (American Type Culture Collection, Manassas, VA, USA) were cultured in RPMI 1640 medium (Gibco, Invitrogen, Carlsbad, CA, USA) supplemented with 10% fetal bovine serum (FBS) and 100 U/mL penicillin-streptomycin (Invitrogen). The retroviral vector pMSCV-neo-IGF-1R containing neomycin resistance gene and expressing human wild-type IGF-1R cDNA was provided by Dr. R. Baserga [24]. IGF-1R-encoding retroviruses were produced by transfection of pMSCV-neo-IGF-1R into Phoenix Amphotropic packaging cells as previously described [25]. MCF7 cells were infected with freshly harvested retroviral supernatant. Two days later infected cells were selected by using 400 μg/mL neomycin. To establish individual positive clones from single cells, the neomycin selected cells were further subjected to limiting dilution in 96-well plates for two-week colony selection. To rule out clonal artefacts, we picked up the wells containing three to five individual positive clones and collected and expanded the multiple clones to generate stable MCF7/IGF-1R cells.

### IGF-1 stimulative exposure

Cells were placed in six-or twelve-well plates at 60% to 70% confluence in regular growth medium. The next day cells were starved overnight with 1% FBS medium. Following an additional two-hour serum deprivation with serum-free medium (SFM), cells were exposed to IGF-1 at a given dose and for a given time course, which was directly followed by cell lysis. To inhibit IGF-1-stimulated signaling, cells were pretreated with a kinase inhibitor in SFM for two hours prior to IGF-1 exposure.

### Cell drug treatments

Cells were seeded with 10,000 cells/well in 96-well plates and incubated overnight. Prior to drug treatments, cells were starved for two days in phenol red-free RPMI 1640 medium (Gibco) supplemented with 5% charcoal/dextran-stripped FBS (CDFBS) (HyClone Laboratories, Thermo Scientific, Logan, UT, USA) devoid of steroid hormones. Starved cells were then subjected to individual or combined drug treatments in triplicate and allowed to proliferate for four days.

### Sulforhodamine B colorimetric assay determining cell proliferation

A sulforhodamine B (SRB) colorimetric assay [26,27] was used to determine cell proliferation that occurred in given drug treatments. In short, drug-treated cells in 96-well plates were fixed with 30 μL of 50% trichloroacetic acid directly added to 100 μL of assay medium per well for 1 hour at 4°C on a shaker, gently washed five times with tap water and air-dried. Fixed cells were stained with 60 μL of 0.4% SRB (dissolved in 1% acetic acid) at room temperature on a shaker for 30 minutes to allow the SRB to bind to protein, rinsed five times with 1% acetic acid to remove unbound dye and then air-dried. Subsequently, the protein-bound SRB in each well was solubilized with 200 μL of 10 mM Tris base solution (pH > 10) for 10 minutes on a plate shaker and measured for its absorbance at 510 nm with a FLUOstar OPTIMA plate reader (BMG LABTECH, Offenburg, Germany). The SRB absorbance values at 510 nm are thus indicative of cell proliferation in response to drug treatments.

### 3D culture

In addition to the SRB assay, a modified 3D culture was established to further examine cellular responses to growth factor IGF-1 stimulation and drug treatments. Briefly, a 96-well plate was coated with 30 μL/well of 100% Matrigel (354230, BD Matrigel™ Basement Membrane Matrix Growth Factor Reduced; BD Biosciences, Franklin Lakes, NJ, USA) at 37°C for 45 minutes. Single cells (10,000 cells/well) were suspended in 120 μL of starving medium (phenol red-free RPMI 1640) containing 2% soluble Matrigel and 5% CDFBS (instead of complete medium) in which E2 was depleted, and the cells were then seeded on top of coating Matrigel. For given treatments, the concentrations of the drugs were adjusted for the total volume of 150 μL. Fresh medium was added every three or four days. After two weeks of culturing on Matrigel, the cells were fixed and stained with 3.7% formaldehyde in phosphate-buffered saline (PBS) containing 0.2% Triton X-100, 0.25 μM rhodamine phalloidin (Sigma) and 1 μg/mL Hoechst blue dye. Confocal imaging of 3D cell culture was performed with a Nikon ECLIPSE TE2000-E (Nikon Instruments, Tokyo, Japan).

### Cell lysis and Western blot analysis

To prepare cell lysates for Western blot analysis, cells were washed three times with ice-cold PBS and lysed on ice for 30 minutes with lysis buffer (50 mM Tris, pH 7.5, 150 mM NaCl, 2 mM ethylenediaminetetraacetic acid, 0.1% sodium dodecyl sulfate (SDS), 1% Nonidet P-40 and 1% deoxycholic acid) freshly supplemented with 100-fold diluted protease inhibitor cocktail (P8340-ML; Sigma-Aldrich). Harvested lysate supernatant was measured for cellular protein concentration using the BCA™ Protein Assay Kit (Thermo Scientific, Rockford, IL, USA). A quantity of 30 μg/lane total protein were separated by SDS-polyacrylamide gel electrophoresis on 7.5% acrylamide gel and electrophoretically transferred to polyvinylidene fluoride membrane (Millipore, Billerica, MA, USA). Prior to primary antibody probe, membrane was blocked for 1 hour at room temperature with 5% bovine serum albumin (BSA) in Tris-buffered saline Tween 20 (TBST) buffer (100 mM Tris, pH 7.4, 500 mM NaCl, 0.05% Tween 20) or with I-Block buffer (Tropix, Applied Biosystems). Phospho-IGF-1Rβ (Tyr1131) and phospho-ERK1/2 (Thr202/Tyr204) were probed in 5% BSA-TBST buffer, whereas phospho-IGF-1Rβ (Tyr1135/Tyr1136) and phospho-Akt (Ser473) were probed in I-Block buffer. HRP-or AP-conjugated secondary antibody incubation was performed in 5% BSA-TBST or I-Block buffer, corresponding to the primary antibodies used. Protein bands were visualized by using the ECL Plus method (GE Healthcare, Little Chalfont, Buckinghamshire, UK), after which the membrane was scanned by using a Typhoon 9400 imager (GE Healthcare) or a Tropix Western-SuperStar™ procedure (Applied Biosystems) by placing the membrane in contact with standard X-ray film (GE Healthcare).

### Immunofluorescence staining

Cells seeded onto coverslips were washed once with PBS, fixed with 80% acetone in H_2_O and then blocked with 5% normal goat serum in PBS containing 0.05% Tween 20. Expression of IGF-1R was detected by mouse monoclonal antibody against IGF-1Rβ and labeled with Alexa Fluor 488 antimouse antibody. Nuclear DNA was stained with 4',6-diamidino-2-phenylindole. The stained cells were visualized under a fluorescence microscope (Nikon Eclipse E600; Nikon Instruments) at a × 60 lens objective.

### Small interfering RNA transfection

To silence target genes in cells, 50 nM SMARTpool siRNA mix (Dharmacon Technologies, Thermo Scientific, Lafayette, CO, USA) was delivered into 15,000 cells/well in 96-well plates by using a standard transfection method with DharmaFECT 4 transfection reagent (Dharmacon Technologies) according to the manufacturer's instructions. After 24 hours, the small interfering RNA (siRNA) transfection mixture was replaced with complete medium or with 5% CDFBS starving medium if drug treatment and SRB proliferation assay were included. Cells were kept in culture for one more day before analysis of knockdown or further treatment.

### Estrogen responsive element-luciferase reporter assay

Cells were resuspended in antibiotic-free culture medium, and 40,000 cells/well were seeded into 48-well plates. By use of Lipofectamine PLUS reagent (Invitrogen), cells were transiently transfected with 0.16 μg of the estrogen response element (ERE)-thymidine kinase-luciferase plasmid kindly provided by Dr. R. Michalides [28]. After three hours, cells were starved with 5% CDFBS medium for two days. Following 12-hour treatments as indicated, cells were washed once with PBS and lysed with 1 × passive lysis buffer (Dual-Luciferase Assay Kit; Promega). ERE-luciferase activity was measured using a luminometer (CentroXS^3 ^LB 960; Berthold Technologies, Bad Wildbad, Germany).

### Statistical analysis

Each average SRB absorbance value was derived from triplicate samples. Statistical analyses of all experimental data were performed using a two-sided Student's *t*-test. Significance was set at *P *< 0.05.

## Results

### Ectopic IGF-1R expression in MCF7/IGF-1R cells enhances IGF-1R tyrosine kinase activity upon IGF-1 ligand stimulation

**Figure 1:**
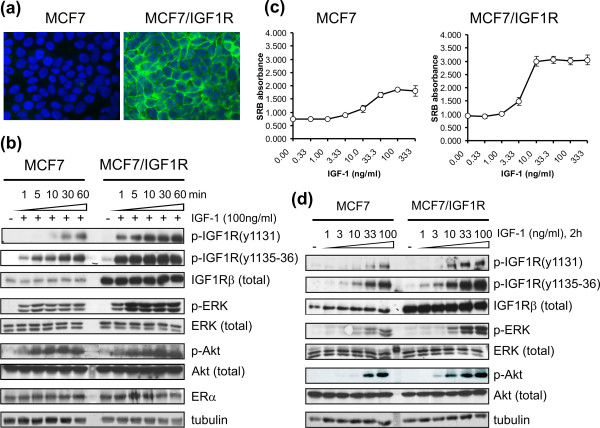
**Characterization of insulin-like growth factor 1 receptor overexpression, autophosphorylation, signaling transduction and proliferation of MCF7/insulin-like growth factor 1 receptor cells in response to insulin-like growth factor 1**. **(a) **Immunofluorescence showing insulin-like growth factor 1 receptor (IGF-1R) overexpression in MCF7/IGF-1R cells compared to parental MCF7 cells. IGF-1R was probed with mouse monoclonal antibody against IGF-1Rβ (green). Cell nuclei were stained with 4',6-diamidino-2-phenylindole (blue). Original magnification, × 60. **(b) **IGF-1R autophosphorylation and signal transduction in time course exposure to IGF-1 (100 ng/mL) for 1, 5, 10, 30 and 60 minutes. ERα, estrogen receptor. **(c) **Proliferative response of MCF7 versus MCF7/IGF-1R cells to IGF-1 stimulation at the dose ranges indicated. Sulforhodamine B (SRB) absorbance values (optical density (OD) 510 nm) are representative of three independent experiments. Data are expressed as means ± SD. **(d) **IGF-1R autoactivation and signal transduction to IGF-1 exposure at various dose levels (1, 3, 10, 33 and 100 ng/mL) for two hours. Total IGF-1R levels were detected by IGF-1Rβ antibody at 97 kDa. IGF-1R triple tyrosine phosphorylation was determined by using phospho-IGF-1Rβ (Tyr1131) and phospho-IGF-1Rβ (Tyr1135/Tyr1136) rabbit antibodies. Phosphorylated extracellular signal-regulated kinase (p-ERK) and phosphorylated protein kinase B (p-Akt) were determined by using p-ERK1/2 and p-Akt antibodies, respectively. The status of the estrogen receptor (ER) was detected by rabbit anti-ERα antibody. Tubulin was detected as a control for protein loading.

To establish a breast cancer cell line stably overexpressing IGF-1R, human MCF7 breast cancer cells were retrovirally transduced with a pMSCV-neo-IGF-1R vector and subjected to single-cloning selection following limiting dilution. The established MCF7/IGF-1R cell line stably expressed ectopic IGF-1R (Figure 1a), with expression approximately 10-fold that of parental MCF7 cells (Figure 1b). The proliferative response of MCF7/IGF-1R cells to IGF-1 (half-maximal effective concentration (EC_50_) = 2.4 ng/mL) was increased compared to MCF7 cells (EC_50 _= 16.0 ng/mL) (Figure 1c).

IGF-1R contains a triple tyrosine cluster in the kinase domain, Tyr1131, Tyr1135 and Tyr1136, which is required for full kinase activation of IGF-1R [29]. To demonstrate IGF-1R autoactivation by ligand binding, time course (Figure 1b) and dose range exposures to IGF-1 (Figure 1d) were performed. Triple tyrosine IGF-1R phosphorylation was initiated rapidly and sustained for long time periods, reaching maximal levels at 100 ng/mL IGF-1. Overall, MCF7/IGF-1R cells displayed stronger IGF-1R autophosphorylation than parental MCF7 cells, indicating that MCF7/IGF-1R cells gained elevated intrinsic IGF-1R tyrosine kinase activity, which is necessary for the activation of the IGF-1-stimulated downstream signaling cascades.

IGF-1R signal transduction involves several major phosphorylation cascades, including the MAPK and PI3K pathways [30,31]. To confirm canonical IGF-1R signal transduction in the cell lines used, the activity of the downstream kinases ERK and Akt was determined. Concurrently with IGF-1R autophosphorylation, both ERK and Akt kinases became phosphorylated in parallel (Figure 1b). While maximal Akt phosphorylation was induced to a similar degree, maximal ERK phosphorylation was clearly higher in MCF7/IGF-1R cells than in MCF7 cells (Figures 1b and 1d), appearing to be consistent with IGF-1R phosphorylation levels. These data indicate that the MAPK/ERK and PI3K/Akt signal transduction cascades are induced via ligand-activated IGF-1R kinase activity, with increased MAPK/ERK signaling in the MCF7/IGF-1R cells.

Of note, equal levels of ERα were detected in both MCF7 and MCF7/IGF-1R cells (Figure 1b), indicating that overexpression and activation of IGF-1R does not influence ERα expression in the MCF7/IGF-1R cell model.

### High levels of IGF-1R signaling render MCF7/IGF-1R cells resistant to the antiestrogens tamoxifen and fulvestrant

**Figure 2:**
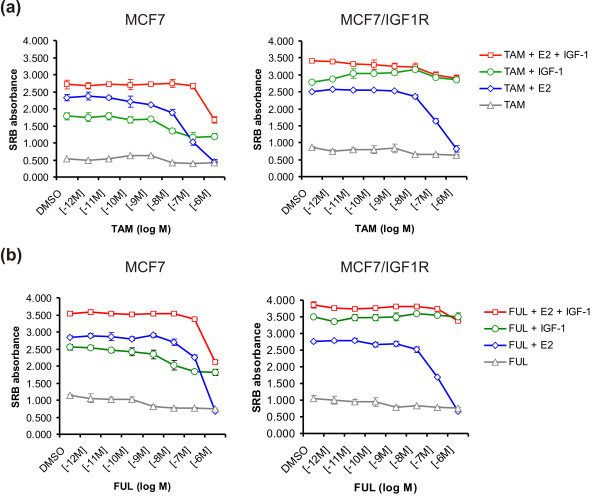
**Antiestrogen resistance of MCF7/IGF-1R cells is induced by high level of IGF-1R signaling**. **(a) **Proliferative behavior of MCF7 versus MCF7/IGF-1R cells in response to 4-hydroxytamoxifen (TAM) in the dose range indicated in combination with estrogen 17β-estradiol (E2) (1 nM), IGF-1 (100 ng/mL) or E2 (1 nM) plus IGF-1 (100 ng/mL). Dimethyl sulfoxide (DMSO) was used as a control. **(b) **Proliferative behavior of MCF7/IGF-1R versus MCF7 cells in response to fulvestrant (FUL) with the same dose range and treatments as were used for TAM. Each treatment was carried out in triplicate. SRB absorbance values are representative of three independent experiments. Data are expressed as means ± SD.

Next, we evaluated the sensitivity of MCF7/IGF-1R cells to E2 and the antiestrogens 4-OH-TAM and FUL. MCF7/IGF-1R cells were responsive to proliferative effects of E2 (EC_50 _= 5 × 10^-11 ^M) (Additional file 1), similar to parental MCF7 cells. The proliferation induced by E2 (1 nM) in both cell types was antagonized by 4-OH-TAM and FUL (IC_50 _= 4 × 10^-8 ^M) (Figure 2), indicating that ectopic IGF-1R expression in MCF7/IGF-1R cells does not affect ERα responses.

To address whether IGF-1 stimulation affects MCF7/IGF-1R cellular sensitivity to antiestrogens, cells were treated with a concentration range of 4-OH-TAM (Figure 2a) or FUL (Figure 2b) in combination with E2 (1 nM), IGF-1 (100 ng/mL) or a combination of E2 and IGF-1 as indicated. While 4-OH-TAM and FUL at 10^-6 ^M completely blocked E2-induced proliferation, IGF-1 stimulation desensitized MCF7/IGF-1R cells to the antiproliferative effects of 4-OH-TAM and FUL, sustaining proliferation level similar to that induced by IGF-1 alone and close to that induced by the IGF-1 and E2 co-treatment. IGF-1 stimulation also led to partial resistance of MCF7 cells to the antiestrogens at 10^-6 ^M, but it was much less significant than that of MCF7/IGF-1R cells. Together, these results indicate that enhanced IGF-1R signaling upon IGF-1 stimulation directly renders MCF7/IGF-1R cells insensitive to 4-OH-TAM and FUL, leading to strong antiestrogen resistance.

### IGF-1R signaling induces antiestrogen resistance of MCF7/IGF-1R cells in an ER-independent manner

**Figure 3:**
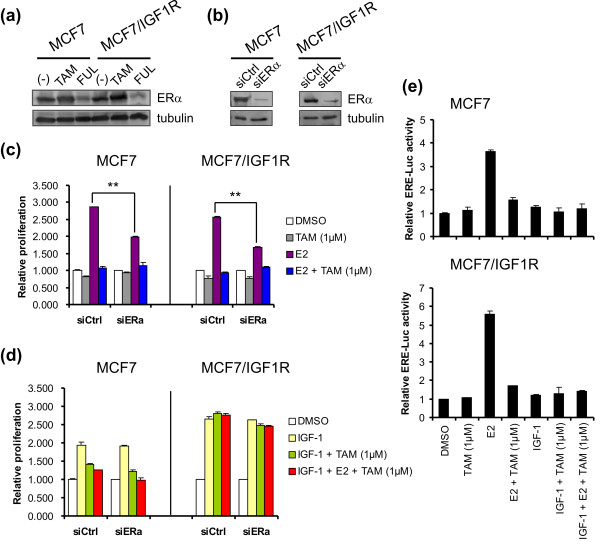
**IGF-1R signal-induced antiestrogen resistance in MCF7/IGF-1R cells is independent of ER status**. **(a) **Differential ER levels in response to antiestrogens TAM and FUL. Cells were treated with 100 nM TAM or FUL for three days following two-day starvation. (-), untreated. **(b) **Knockdown of ER expression by small interfering RNA (siRNA). **(c) **Effect of ERα knockdown by siRNA on cell proliferation in response to TAM (1 μM), E2 (1 nM) or E2 (1 nM) plus TAM (1 μM). siCtrl, siRNA control; siERa, siRNA ERα; **P < 0.01. **(d) **Effect of ERα knockdown on cell proliferation in response to IGF-1 (100 ng/mL), IGF-1 (100 ng/mL) plus TAM (1 μM) or a combination of IGF-1 (100 ng/mL), E2 (1 nM) and TAM (1 μM). SRB proliferation OD values were normalized to the control DMSO. Data are representative of three individual experiments. Data are expressed as means ± SD. **(e) **Estrogen response element (ERE)-dependent luciferase (Luc) activity of MCF7/IGF-1R cells in the presence of TAM (1 μM), E2 (1 nM), E2 (1 nM) plus TAM (1 μM) and a combination of IGF-1 (100 ng/mL), E2 (1 nM) and TAM (1 μM). Luciferase activity was normalized to DMSO control. Three individual ERE-luciferase assays were performed. Error bars represent ± SD.

4-OH-TAM and FUL regulate ER function differently. 4-OH-TAM is a competitive E2 antagonist and blocks ER transcriptional activity [32], whereas FUL antagonizes E2 and degrades ER protein [33]. This differential ER regulation by 4-OH-TAM and FUL was equal in both MCF7 and MCF7/IGF-1R cells. 4-OH-TAM did not affect ERα expression, while FUL decreased ERα protein levels (Figure 3a).

To investigate whether tamoxifen resistance of MCF7/IGF-1R cells is related to ER function, we silenced ERα by siRNA transfection, reaching > 80% knockdown of ERα (Figure 3b). Compared to siRNA control (siCtrl), ERα silencing (siERα) significantly inhibited proliferation by E2 (65% to 70%; *P *< 0.01) (Figure 3c), but not by IGF-1 either alone or in cotreatment with E2 plus 4-OH-TAM (1 μM) (Figure 3d). In addition, the ERE-luciferase assay showed that IGF-1 did not affect ERE-mediated transcription (Figure 3e). While E2 led to a high level of ERE-mediated transcription, 4-OH-TAM sustained its antagonistic action on E2 and suppressed ER transcriptional ability in the presence of IGF-1 (Figure 3e). Furthermore, mRNA expression analysis demonstrated that IGF-1 stimulation did not significantly interfere with the expression of ER target genes, for instance, the known E2-responsive genes *CXCL12 *(chemokine (C-X-C motif) ligand 12; also named stromal cell-derived factor 1) and *FOXC1 *(forkhead box C1) [34,35]. The induction of both *CXCL12 *(Additional file 2a) and *FOXC1 *(Additional file 2b) gene expression in response to E2 was significantly inhibited by 4-OH-TAM antagonistic activity in either the absence or the presence of IGF-1.

These results suggest that IGF-1R signal-mediated antiestrogen resistance in MCF7/IGF-1R cells does not involve ERα-dependent processes. IGF-1-stimulated proliferation may run parallel to E2-induced proliferation and therefore is not inhibited by the antiestrogens 4-OH-TAM and FUL.

### IGF-1R signaling involves the MAPK/ERK and PI3K/Akt pathways to mediate antiestrogen resistance of MCF7/IGF-1R cells

**Figure 4:**
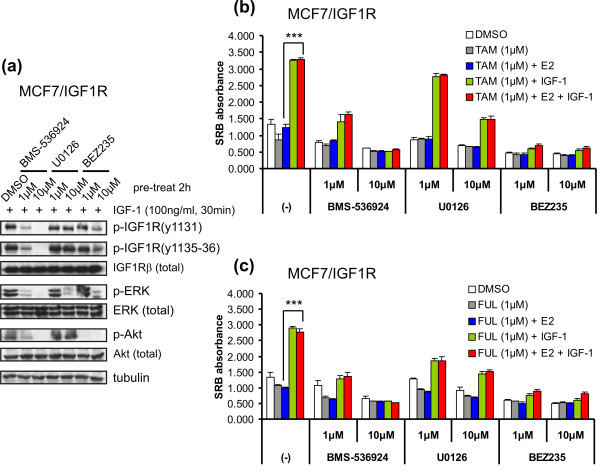
**Involvement of mitogen-activated protein kinase/extracellular signal-regulated kinase and phosphatidylinositol 3-kinase/Akt in IGF-1R signal-mediated antiestrogen resistance of MCF7/IGF-1R cells**. **(a) **Inhibitory effects of kinase inhibitors on IGF-1R autoactivation and its signaling transduction. **(b) **Inhibitory effects of kinase inhibitors on tamoxifen resistance of MCF7/IGF-1R cells in response to TAM (1 μM) individually or combined with E2 (1 nM) and IGF-1 (100 ng/mL) as indicated. **(c) **Inhibitory effects of kinase inhibitors on FUL resistance of MCF7/IGF-1R cells in response to FUL (1 μM) individually or combined with E2 (1 nM) and IGF-1 (100 ng/mL) as indicated. (-), no kinase inhibitor. SRB absorbance values are representative of three independent experiments. Error bars represent ± SD. ***P < 0.001.

Activation of IGF-1R signaling upon IGF-1 exposure led to downstream ERK and Akt phosphorylation (Figures 1b and 1c). To evaluate the role of these kinase pathways in IGF-1R signal-induced antiestrogen resistance in MCF7/IGF-1R cells, we first used several specific kinase inhibitors, including the IGF-1R inhibitor BMS-536924 [36], the MEK inhibitor U0126 [37] and the PI3K inhibitor BEZ235 [38]. BMS-536924 efficiently blocked IGF-1R autophosphorylation by IGF-1 stimulation and downstream phosphorylation of both ERK and Akt by IGF-1R signal transduction (Figure 4a). U0126 (10 μM) largely inhibited ERK phosphorylation without interfering with activation of either IGF-1R or Akt. BEZ235 (1 μM) completely blocked Akt phosphorylation, leaving IGF-1R and ERK signaling intact (Figure 4a). These results suggest that, indeed, ERK and Akt are downstream from IGF-1R signaling in a linear fashion.

Next, the engagement of the ERK and Akt pathways in IGF-1R signal-mediated antiestrogen resistance in MCF7/IGF-1R cells was examined by use of the respective kinase inhibitors (Figures 4b and 4c). IGF-1 stimulation increased MCF7/IGF-1R proliferation by 60% to 64% (*P *< 0.001) over the cotreatment of 4-OH-TAM or FUL (1 μM) with E2, whereas this IGF-1-promoted antiestrogen resistance was significantly abrogated by BMS-536924, U0126 and BEZ235 equivalently to their inhibitory effects on relative kinase phosphorylation.

**Figure 5:**
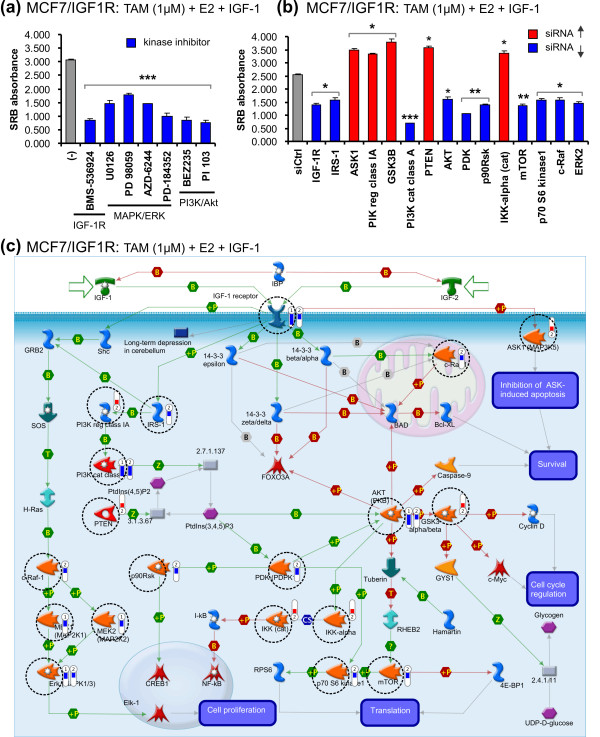
**The key IGF-1R signaling components in the regulation of TAM resistance of MCF7/IGF-1R cells**. **(a) **Selected kinase inhibitors inhibiting IGF-1R signal-mediated TAM resistance. **(b) **Selected siRNA targeting genes (as named) involved in IGF-1R signal-mediated TAM resistance. Concentrations used: TAM (1 μM), E2 (1 nM) and IGF-1 (100 ng/mL). (-), no kinase inhibitor. siCtrl, nontargeting siRNA. Results are representative of duplicate experiments. Data are expressed as means ± SD. *P < 0.05. **P < 0.01. ***P < 0.001. **(c) **MetaCore Pathway Analysis showing the functional activities of the key players (dashed circles) in IGF-1R signal-mediated TAM resistance. Fold changes in cell proliferation as the result of inhibition of selected kinase inhibitors or knockdown of target genes are given in Additional file 3. 1, experiment 1 (exp 1) with kinase inhibitors. 2, experiment 2 (exp 2) with siRNA. Red bar, increase in proliferation. Blue bar, decrease in proliferation.

The key components involved in the PI3K/Akt and MAPK/ERK pathways have been well defined [39]. Using several other specific kinase inhibitors (Figure 5a) and targeting siRNA (Figure 5b and Additional file 3), we further delineated the functional activities of the known PI3K/Akt and MAPK/ERK components in the 4-OH-TAM resistance of MCF7/IGF-1R cells and positioned them in the IGF-1R signaling map (Figure 5c, dashed circles) by using MetaCore Pathway Analysis software (GeneGo, St. Joseph, MI, USA) according to fold changes in proliferation (Additional file 3 Table S1). As expected, inhibition of IGF-1R by either kinase inhibitor or siRNA silencing blocked IGF-1-stimulated proliferation (Figure 5c, exp 1 and 2, blue bars). Insulin receptor substrate 1 (IRS-1), which is known to be phosphorylated by autoactivated IGF-1R upon ligand binding, was shown to be involved in IGF-1R-mediated 4-OH-TAM resistance (Figure 5c, exp 2, blue bar). Kinase inhibitors blocking the signaling of the MAPK/ERK and PI3K/Akt pathways led to overall inhibition of proliferation (Figure 5c, blue bars in exp 1). Silencing of the major MAPK/ERK and PI3K/Akt kinases c-Raf-1 (*Raf1*), ERK2 (*MAPK1*), PI3K cat class A (*PIK3CA*), Akt (*AKT1*), mTOR (*FRAP1*) and p70 S6 kinase 1 (*RPS6KB1*) largely abrogated IGF-1-induced 4-OH-TAM resistance (Figure 5c, exp 2, blue bars). The PI3K negative regulators PI3K reg class IA (*PI3KR1*) and phosphatase and tensin homolog (*PTEN*) greatly increased proliferation when knocked down (Figure 5c, exp 2, red bars), further indicating the role of the PI3K pathway in the 4-OH-TAM resistance of MCF7/IGF-1R cells. In a novel way, our results reveal that, when targeted by siRNA, phosphoinositide-dependent kinase (PDK) (*PDPK1*) and p90Rsk (*RPS6KA2*), which are known positive regulators of transcription factor CREB1 (cyclic adenosine monophosphate-responsive element binding protein 1), decreased cell proliferation (Figure 5c, exp 2, blue bars), whereas inhibitor of nuclear factor κB kinase subunit (IKK) (*CHUK*) and glycogen synthase kinase 3 (GSK3) (*GSK3B*), which are known inhibitors of transcription factors NF-κB and c-Myc, significantly increased cell proliferation (Figure 5c, exp 2, red bars). In addition, silencing of apoptosis signal-regulating kinase 1 (ASK1) (*MAP3K5*), which is negatively regulated by IGF-1R phosphorylation to prevent apoptosis, enhanced cell proliferation (Figure 5c, exp 2, red bars).

Together, our results confirm that IGF-1R signaling incites multiple downstream cascades in which the key MAPK/ERK and PI3K/Akt components constitute the central signaling nodes that regulate IGF-1/IGF-1R signal-mediated proliferation and antiestrogen resistance in MCF7/IGF-1R cells.

### Tamoxifen resistance of IGF-1-stimulated MCF7/IGF-1R cells is induced in 3D culture

**Figure 6:**
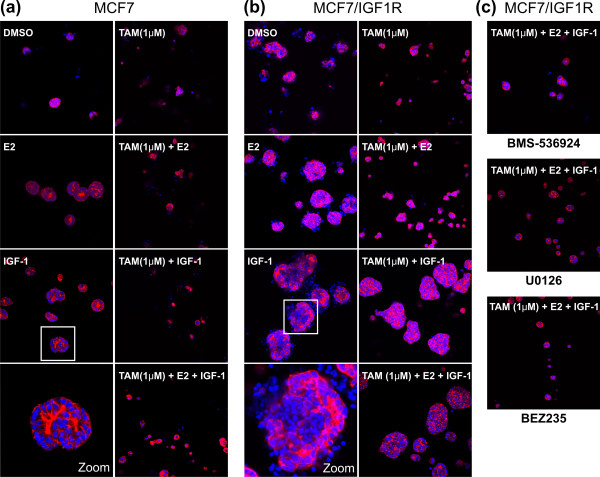
**Three-dimensional responses of MCF7/IGF-1R cells to TAM (1 μM), E2 and IGF-1**. Compared to parental MCF7 cells **(a)**, MCF7/IGF-1R cells **(b) **in three-dimensional (3D) culture formed bigger acini in response to IGF-1 stimulation and displayed significant TAM resistance when treated with TAM (1 μM) + E2 + IGF-1, which was removable by kinase inhibitors BMS-536924, U0126 and BEZ235 **(c)**. Cells (10,000/well) were seeded in 96-well plates. Acini were formed on 100% Matrigel and cultured for 14 days in starving medium containing 2% Matrigel and 5% charcoal/dextran-stripped fetal bovine serum with the treatments as indicated. Concentrations used: TAM (1 μM), E2 (1 nM) and IGF-1 (100 ng/mL). Confocal image original magnification, × 20. Red, rhodamine phalloidin (actin). Blue, Hoechst blue stain. Results are representative of two individual experiments.

Next, we further investigated the effect of IGF-1 stimulation on MCF7/IGF-1R 4-OH-TAM resistance in a more structurally and physiologically relevant context by use of a modified 3D culture. Both parental MCF7 (Figure 6a) and MCF7/IGF-1R cells (Figure 6b) were responsive to E2 or IGF-1 by forming acini on Matrigel, but the response was significantly larger in MCF7/IGF-1R cells with altered acinar morphogenesis (zoom images at bottom of Figures 6a and 6b). 4-OH-TAM effectively blocked E2-induced acini in both cell types (4-OH-TAM + E2). However, addition of IGF-1 (4-OH-TAM + E2 + IGF-1) overcame the antiproliferative effects of 4-OH-TAM and allowed acini growth of MCF7/IGF-1R cells but not MCF7 cells, demonstrating IGF-1-stimulated 4-OH-TAM resistance of MCF7/IGF-1R cells in 3D culture. Similarly to the SRB 2D assay, inhibition of IGF-1R/ERK/Akt signaling by respective kinase inhibitors restored 4-OH-TAM sensitivity of MCF7/IGF-1R cells in 3D culture (Figure 6c).

### At low concentrations, tamoxifen displays an agonistic behavior in IGF-1-stimulated MCF7/IGF-1R cells

**Figure 7:**
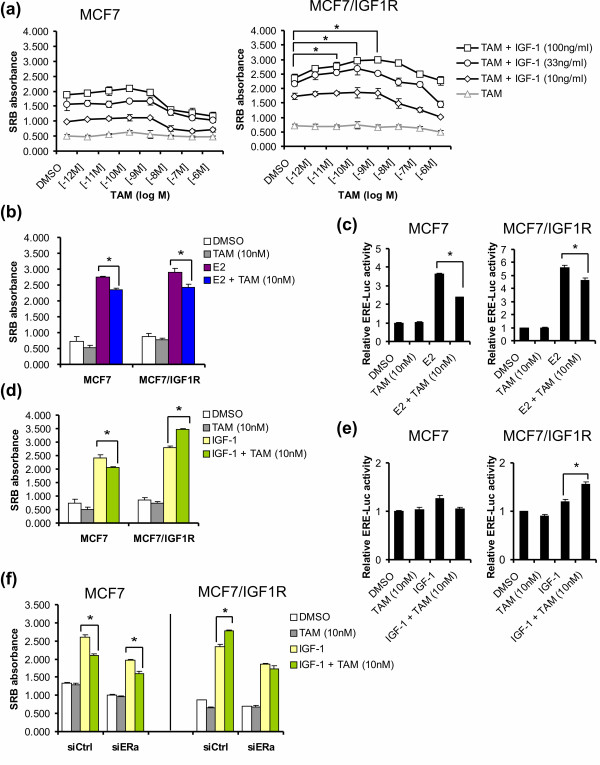
**TAM (10 nM) acts as an agonist in MCF7/IGF-1R stimulated by IGF-1**. **(a) **Dose dependence of TAM resistance on IGF-1. **(b) **Antagonistic effect of 10 nM TAM on 1 nM E2-induced cell proliferation. **(c) **Antagonistic effect of 10 nM TAM on 1 nM E2-induced ERE-Luc activity. **(d) **Agonistic effect of 10 nM TAM on 100 ng/mL IGF-1-stimulated MCF7/IGF-1R cells. **(e) **ERE-Luc activity induced by agonistic TAM (10 nM) in IGF-1-stimulated MCF7/IGF-1R cells. **(f) **ER dependence of agonistic effect of TAM (10 nM) in MCF7/IGF-1R cells. siCtrl, siRNA control; siERα, siRNA ERα. Results are representative of three individual experiments. Luciferase activity was normalized to DMSO control. Error bars represent ± SD. *P < 0.05.

We have demonstrated that IGF-1 treatment of MCF7/IGF-1R cells overruled the antiproliferative effect of tamoxifen (at 1 μM). However, we also note that at low concentrations of 4-OH-TAM, an increase in proliferation was triggered on top of IGF-1-driven proliferation (Figure 2a), suggesting a potential role for enhanced intrinsic IGF-1R signaling in tamoxifen resistance. To investigate to what extent IGF-1R signaling causes this 4-OH-TAM agonistic effect, cells were coexposed to 4-OH-TAM in doses of 10, 33 or 100 ng/mL IGF-1, which were shown to cause different levels of sustained receptor and downstream signaling activation (Figure 1d). Consistently, IGF-1 promoted a 4-OH-TAM promitogenic effect in MCF7/IGF-1R cells but not in MCF7 cells, with the highest potential occurring at 4-OH-TAM 10^-9 ^to 10^-8 ^M (Figure 7a). This 4-OH-TAM promitogenic effect was most strongly induced by IGF-1 between 33 and 100 ng/mL, concentrations which were shown to induce maximal IGF-1R signaling (Figure 1d).

As expected, at 10 nM 4-OH-TAM, a marginal antagonistic effect was observed to block E2 (1 nM)-mediated proliferation (Figure 7b) and ERE-luciferase activity (Figure 7c) in both MCF7 and MCF7/IGF-1R cells. Interestingly, 10 nM 4-OH-TAM provided a mild promitogenic effect, increasing IGF-1-dependent proliferation (17% to 27%; *P *< 0.05) (Figure 7d) and ERE-mediated ER transcriptional activity in MCF7/IGF-1R cells (*P *< 0.05) (Figure 7e), whereas it remained inhibitory in parental MCF7 cells (15% to 20%; *P *< 0.05) (Figures 7d and 7e). These agonistic effects were not observed for FUL.

To further evaluate whether ERα is functionally required for this 4-OH-TAM agonistic switch in MCF7/IGF-1R cells, siRNA ERα knockdown experiments were performed. In siRNA control (siCrtrl) conditions, 10 nM 4-OH-TAM maintained a promitogenic effect on IGF-1-stimulated MCF7/IGF-1R cells (18% to 25%; *P *< 0.05), but ERα knockdown (siERα) eliminated the proliferation increase induced by 10 nM 4-OH-TAM (Figure 7f). These results imply that enhanced IGF-1R intrinsic kinase activity triggered this switch of 4-OH-TAM (10 nM) from an ERα antiproliferative modulator into a promitogenic modulator, indicating an agonistic effect on ER.

### Tamoxifen agonistic behavior in MCF7/IGF-1R cells can be attributed to the MAPK/ERK pathway

**Figure 8:**
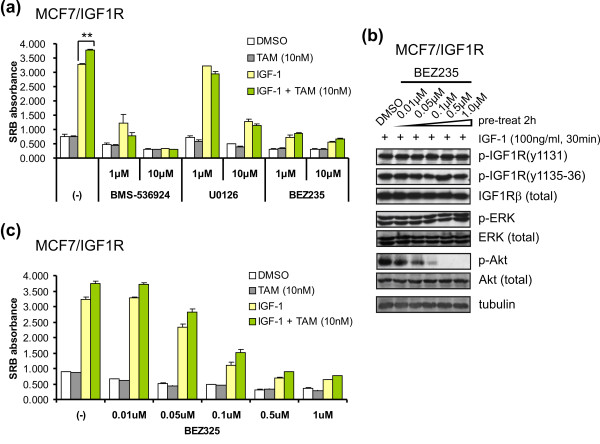
**TAM (10 nM) agonistic behavior in MCF7/IGF-1R cells is not inhibited by phosphatidylinositol 3-kinase/Akt inhibitor BEZ235**. **(a) **Inhibitory effects of kinase inhibitors BMS-536924, U0126 and BEZ235 on agonistic effect of TAM (10 nM) in the presence of IGF-1 (100 ng/mL). **P < 0.01. **(b) **Inhibitory effects of phosphatidylinositol 3-kinase (PI3K)/Akt inhibitor BEZ235 at various dose ranges on IGF-1R signaling. **(c) **Agonistic behavior of TAM (10 nM) in response to BEZ235 kinase inhibitor. (-), no BEZ235. Results are representative of three independent experiments. Data are expressed as means ± SD.

We also investigated whether the agonistic behavior of 4-OH-TAM (10 nM) in IGF-1-stimulated MCF7/IGF-1R cells is conferred via the ERK and Akt signaling pathways. Inhibition of IGF-1R signaling by BMS-536924 effected complete abrogation of IGF-1-stimulated proliferation and 4-OH-TAM (10 nM) agonistic action (Figure 8a). While 1 μM U0126 inhibited neither phosphorylation of ERK (Figure 4a) nor IGF-1-stimulated proliferation, it did clearly attenuate tamoxifen agonistic behavior (*P *< 0.01), with a subsequent competence in proliferative inhibition at 10 μM (Figure 8a). IGF-1-stimulated proliferation was drastically restrained by BEZ235 at either 1 or 10 μM (Figure 8a). Similar inhibitory patterns by the kinase inhibitors were observed in parental MCF7 cells, except for the 4-OH-TAM (10 nM) promitogenic effect (Additional file 4a).

We noted that although the BEZ235 inhibitory effect (even at 1 μM) was drastic, the 4-OH-TAM (10 nM) agonistic action seemed not to be completely abolished (Figure 8a). Therefore, further experiments with a lower dose range of BEZ235 were performed. While IGF-1R and ERK signaling remained unaffected in response to IGF-1, the phosphorylation level of Akt was diminished with an increase in the dose of BEZ235, largely at 0.1 μM and entirely at 0.5 μM (Figure 8b). Likewise, the cell proliferation rate decreased correspondingly to Akt phosphorylation levels, significantly dropping at 0.1 μM BEZ235 (Figure 8c), which occurred similarly in parental MCF7 cells (Additional file 4b). Intriguingly, the 4-OH-TAM (10 nM) agonistic effect on MCF7/IGF-1R cells appeared irreversible by BEZ235 (Figure 8c). These data suggest that 4-OH-TAM (10 nM) agonist conversion results from elevated intrinsic IGF-1R/MAPK/ERK signaling in MCF7/IGF-1R cells.

## Discussion

The underlying mechanisms for antiestrogen resistance are multifactorial and poorly understood. Studies of growth factor receptor-mediated resistance mechanisms have mainly focused on the RTK signaling by EGFR and HER2 [1,5,7,22,40]. Our data based on the MCF7/IGF-1R cell model demonstrate that, more than just a modulatory role, IGF-1R conveys its own ligand receptor signaling to drive resistance to tamoxifen and FUL. IGF-1R has been shown to facilitate breast cancer survival, migration, invasion and metastasis [8-12]. Herein we have shown that there is a causative mechanism of the IGF-1/IGF-1R axis in the antiestrogen drug resistance of breast cancer cells. IGF-1R signal-mediated tamoxifen resistance in 3D culture indicates the likelihood that these findings can be extended to the *in vivo *situation.

Clinical studies have revealed that phosphorylation of IGF-1R is correlated with poor outcomes of breast cancers, whereas total IGF-1R level is not [15]. Our data show that enhanced RTK activity of IGF-1R is required for the antiestrogen-resistant phenotype of MCF7/IGF-1R cells and that IGF-1R does not elicit antiresistance in the presence of the IGF-1R inhibitor BMS-536924. We propose that elevated IGF-1R signaling can be an important element of breast cancer antiestrogen resistance.

Resistance to antiestrogens may occur *de novo *in initial endocrine therapy or may be acquired later, after an early response. Antiestrogen-resistant breast cancer cells increase their utilization of nongenomic signaling pathways, while genomic ER action seems less active, since in most cases there is no loss of ER when resistance to endocrine therapy develops [18,40-42]. Several successive studies have shown that changes in ER levels do not underlie antiestrogen resistance of breast cancers [43-46]. Other studies have reported that overexpression of IGF-1R or its major substrate, IRS-1, in ER-positive breast cancer cells augments cell proliferation and reduces estrogen growth dependence [47-49]. In addition, phosphorylation of IGF-1R in human breast cancers has no correlation with ER expression [15]. In support of these findings, we have shown that overexpression and phosphorylation of IGF-1R in antiestrogen-resistant MCF7/IGF-1R cells affected neither ER protein levels nor their transcriptional activity in response to estrogen and antiestrogens. In E2-depleted starving medium, exposure of MCF7/IGF-1R cells to IGF-1 (100 ng/mL) abolished the E2 requirement for cell growth and resulted in resistance to antiestrogens (1 μM). Importantly, ERα knockdown by siRNA did not alleviate IGF-1R signal-mediated tamoxifen resistance in MCF7/IGF-1R cells, and ERα degradation by FUL did not rescue the resistant phenotype either. Furthermore, ERE reporter assay and mRNA expression analysis demonstrated that E2-induced ER transcriptional activity and expression of the ER target genes *CXCL12 *and *FOXC1 *are inhibited by 4-OH-TAM in the presence or absence of IGF-1. Thus, our data indicate that the IGF-1/IGF-1R signaling route can incite antiestrogen resistance of MCF7/IGF-1R cells, without cross-talk with the ERα pathway at the moment of IGF-1 ligand stimulation. Therefore, we emphasize that IGF-1R signaling may act as a crucial contributor to antiestrogen resistance in breast cancer, independently of E2 and regardless of ER status.

We have determined that upon IGF-1 ligand stimulation, IGF-1R conveys its signal via the MAPK/ERK and PI3K/Akt pathways, the major downstream pathways integrating signals from RTKs, to mediate antiestrogen resistance in MCF7/IGF-1R cells. Blockage of either pathway by various kinase inhibitors restores cell sensitivity to the antiestrogens tamoxifen and FUL. Specific siRNA silencing of IGF-1R's substrate, IRS-1, and the key MAPK/ERK and PI3K/Akt kinase components confirms their individual roles in the IGF-1R signal-mediated 4-OH-TAM resistance of MCF7/IGF-1R cells and implies roles for the oncogene *PIK3CA*, the tumor suppressor *PTEN*, the apoptotic modulator ASK1 (*MAP3K5*) and transcription factor regulators such as PDK (*PDPK*), p90Rsk (*RPS6KA2*), IKK (*CHUK*) and GSK3 (*GSK3B*). These results suggest that targeting either IGF-1R itself or downstream MAPK/ERK and PI3K/Akt signaling components may resensitize breast cancer cells to antiestrogen tamoxifen and FUL.

Tamoxifen can have both agonist and antagonist properties [18,50,51]. Herein we have shown that 4-OH-TAM switches from an antagonist to an agonist in IGF-1-stimulated MCF7/IGF-1R cells. Interestingly, this effect was primarily observed at a low concentration of 4-OH-TAM (10 nM) and was associated with mild ERE-mediated ER transcriptional activity. Knockdown of ERα abrogated this agonistic effect of 4-OH-TAM. In addition, this agonistic 4-OH-TAM (10 nM)-promoted proliferation appeared to be dependent on increased MAPK/ERK signaling. Phosphorylation levels of ERKs were increased in IGF-1-stimulated MCF7/IGF-1R cells, significantly higher than those in parental MCF7 cells, whereas Akt phosphorylation remained similar. These results are congruent with those of an earlier study which presented the same activation patterns of ERK and Akt downstream of IGF-1/IGF-1R signaling [49]. Other studies have shown that in acquired tamoxifen-resistant cells or MCF7/HER2 cells, enhanced EGFR and HER2 expression increases MAPK/ERK signaling, leading to agonist activity of tamoxifen, but Akt remains at equal levels [18,42,52-54]. Particularly in tamoxifen-resistant cells, tamoxifen at 100 nM acts as an agonist by increasing proliferation and even triggers ERK phosphorylation as quickly as E2 does [55]. Our data indicate that the tamoxifen agonistic properties can be induced by enhanced activation of IGF-1R signaling and suggest that an increase in MAPK/ERK cascades downstream of IGF-1R RTK signaling may be essential for tamoxifen agonism at low concentrations.

## Conclusions

Our findings using the MCF7/IGF-1R breast cancer cell line model provide evidence that elevated IGF-1R signaling determines the sensitivity of breast cancer cells to antiestrogens and further define the role of the IGF-1/IGF-1R axis in the pleiotropic mechanisms of breast cancer antiestrogen resistance. The MCF7/IGF-1R cell line represents a useful model for investigating the critical elements in IGF-1/IGF-1R-driven proliferation and antiestrogen resistance.

## Abbreviations

Akt: protein kinase B; AP: alkaline phosphatase; ASK1: apoptosis signal-regulating kinase 1; Bcl-2: B-cell lymphoma 2; Bcl-xL: B-cell lymphoma extra large; BSA: bovine serum albumin; CDFBS: charcoal/dextran-stripped fetal bovine serum; *CXCL12*: chemokine (C-X-C motif) ligand 12 chemokine ligand 12 (stromal cell-derived factor 1); DAPI: 4',6-diamidino-2-phenylindole; DMSO: dimethyl sulfoxide; E2: estrogen 17β-estradiol; EC_50_: half-maximal effective concentration; EGFR: epidermal growth factor receptor; ER: estrogen receptor; ERE: estrogen response element; ERK: extracellular signal-regulated kinase; FBS: fetal bovine serum; *FOXC1*: forkhead box C1; FUL: fulvestrant; GSK3: glycogen synthase kinase 3; HER2: human epidermal growth factor receptor 2; HRP: horseradish peroxidase; IC_50_: half-maximal inhibitory concentration; IGF-1: insulin-like growth factor 1; IGF-1R: insulin-like growth factor 1 receptor; IKK: inhibitor of nuclear factor κB kinase subunit; IRS-1: insulin receptor substrate 1; MAPK: mitogen-activated protein kinase; MEK: mitogen-activated protein kinase kinase; mTOR: mammalian target of rapamycin; Myc: myelocytomatosis viral oncogene homolog; NF-κB: nuclear factor κB; 4-OH-TAM: 4-hydroxytamoxifen; PBS: phosphate-buffered saline; PDK: phosphoinositide-dependent kinase; PI3K: phosphatidylinositol 3-kinase; PTEN: phosphatase and tensin homolog; RTK: receptor tyrosine kinase; SFM: serum-free medium; siRNA: small interfering RNA; SRB: sulforhodamine B.

## Competing interests

The authors declare that they have no competing interests.

## Authors' contributions

YZ, JM and BvdW designed the research and wrote the manuscript. YZ and MM performed the majority of the experiments. YZ, SR and LP established and modified the 3D Matrigel assay. HdB helped with 3D confocal imaging. All authors read and approved the final manuscript.

## Supplementary Material

Additional file 1 **Figure S1. Responsiveness of MCF7 versus MCF7/insulin-like growth factor 1 receptor (IGF-1R) cells to 17β-estradiol (E2)**. The sulforhodamine B (SRB) data shown are representative of three individual experiments. Data are expressed as means ± SD. Click here for file

Additional file 2 **Figure S2. Expression levels of E2-responsive genes *CXCL12 *and *FOXC1 *in the presence of tamoxifen, E2 and IGF-1**. For microarray analysis of gene expression, MCF7/IGF-1R cells were seeded at 60% confluence in 6-cm plates and subjected to three-day starvation in 5% charcoal/dextran-stripped fetal bovine serum medium prior to treatments with 4-hydroxytamoxifen (TAM) (10 μM), E2 (10 nM) and IGF-1 (100 ng/mL) as indicated. Each treatment was performed in triplicate. After 6 hours of treatment, cells were harvested and total RNA was extracted using an RNA isolation kit (Ambion, Inc., Austin, TX, USA). RNA quality and integrity were assessed by using the Agilent 2100 Bioanalyzer System (Agilent Technologies, Santa Clara, CA, USA). The Affymetrix 3' IVT Express Kit (Affymetrix, Santa Clara, CA, USA) was used to synthesize biotin-labeled cRNA, and this was hybridized to an Affymetrix HG-U133 PM Array plate reader. Raw expression data were obtained by probe summarization and background correction according to the robust multiarray averaging method [56]. Median normalization of raw expression data and identification of differentially expressed genes using a random variance *t*-test was performed using BRB-ArrayTools [57] version 4.1.0 Beta 2 Release (developed by Dr. Richard Simon and BRB-ArrayTools Development Team members). Expression levels of the E2-responsive genes *CXCL12 *and *FOXC1 *were normalized to their levels in control and dimethyl sulfoxide-treated cells. Data are expressed as means ± SD. Click here for file

Additional file 3 **Table S1**. Effects of various kinase inhibitors and siRNA on IGF/E2/TAM-induced cell proliferation of MCF7/IGF-1R cells. Click here for file

Additional file 4 **Figure S3**. **(a) **Inhibitory effects of kinase inhibitors BMS-536924, U0126 and BEZ235 on MCF7 cells in response to TAM (10 nM) and IGF-1 (100 ng/mL) as indicated. **(b) **Inhibitory effects of kinase inhibitor BEZ35 at a dose range on cell proliferation of MCF7 cells in response to TAM (10 nM) and IGF-1 (100 ng/mL) as indicated. Original data are representative of three independent experiments. Data are expressed as means ± SD. Click here for file
