# Supplementary figures and images for: Elevated insulin-like growth factor 1 receptor signaling induces antiestrogen resistance through the MAPK/ERK and PI3K/Akt signaling routes
Source: Breast Cancer Res. 2011 May 19;13(3):R52. doi: 10.1186/bcr2883 (PMC3218939; doi:10.1186/bcr2883)

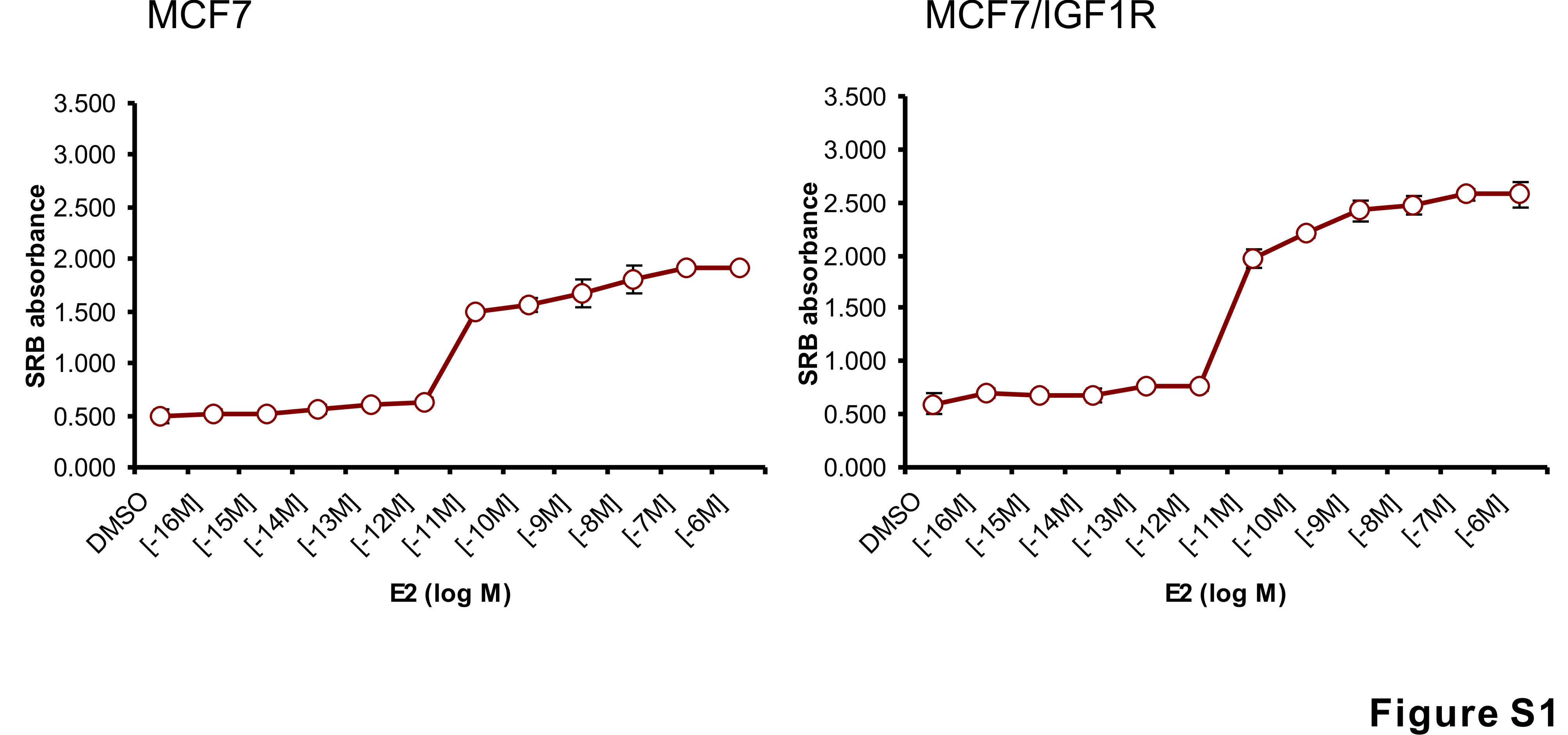

Supplement: Additional file 1 — Figure S1. Responsiveness of MCF7 versus MCF7/insulin-like growth factor 1 receptor (IGF-1R) cells to 17β-estradiol (E2). The sulforhodamine B (SRB) data shown are representative of three individual experiments. Data are expressed as means ± SD. [file bcr2883-S1.JPEG]

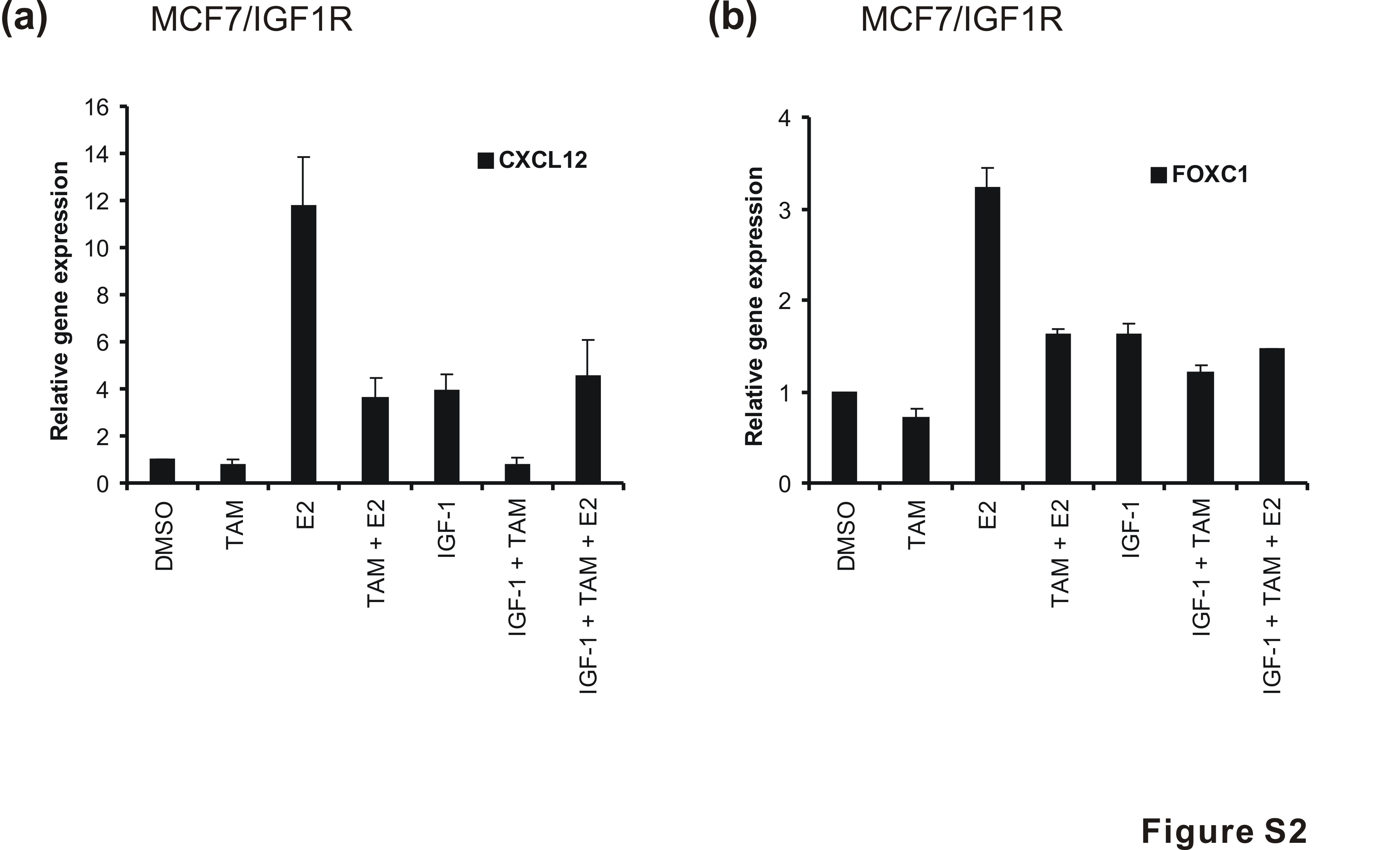

Supplement: Additional file 2 — Figure S2. Expression levels of E2-responsive genes CXCL12 and FOXC1 in the presence of tamoxifen, E2 and IGF-1. For microarray analysis of gene expression, MCF7/IGF-1R cells were seeded at 60% confluence in 6-cm plates and subjected to three-day starvation in 5% charcoal/dextran-stripped fetal bovine serum medium prior to treatments with 4-hydroxytamoxifen (TAM) (10 μM), E2 (10 nM) and IGF-1 (100 ng/mL) as indicated. Each treatment was performed in triplicate. After 6 hours of treatment, cells were harvested and total RNA was extracted using an RNA isolation kit (Ambion, Inc., Austin, TX, USA). RNA quality and integrity were assessed by using the Agilent 2100 Bioanalyzer System (Agilent Technologies, Santa Clara, CA, USA). The Affymetrix 3' IVT Express Kit (Affymetrix, Santa Clara, CA, USA) was used to synthesize biotin-labeled cRNA, and this was hybridized to an Affymetrix HG-U133 PM Array plate reader. Raw expression data were obtained by probe summarization and background correction according to the robust multiarray averaging method [56]. Median normalization of raw expression data and identification of differentially expressed genes using a random variance t-test was performed using BRB-ArrayTools [57] version 4.1.0 Beta 2 Release (developed by Dr. Richard Simon and BRB-ArrayTools Development Team members). Expression levels of the E2-responsive genes CXCL12 and FOXC1 were normalized to their levels in control and dimethyl sulfoxide-treated cells. Data are expressed as means ± SD. [file bcr2883-S2.JPEG]

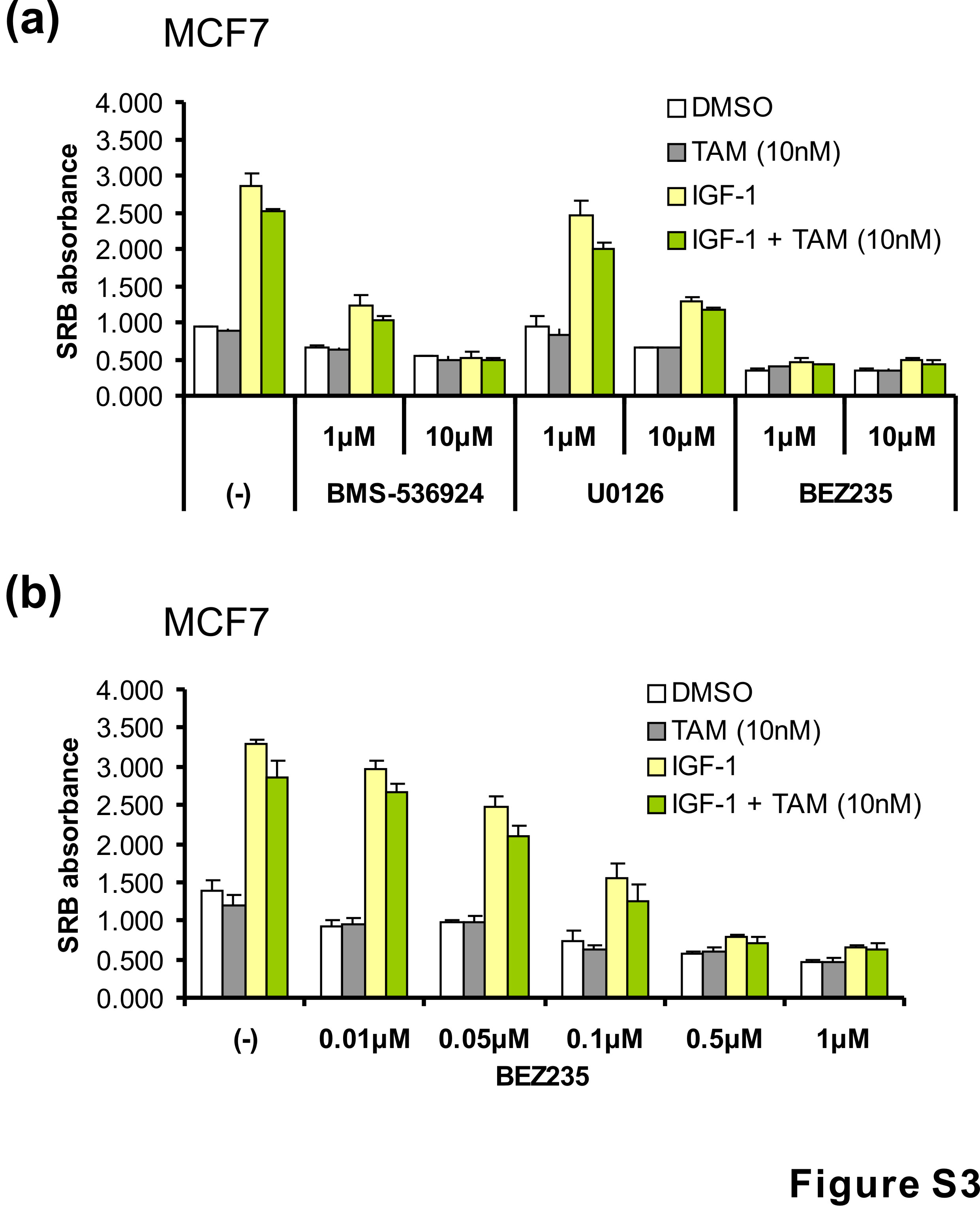

Supplement: Additional file 4 — Figure S3. (a) Inhibitory effects of kinase inhibitors BMS-536924, U0126 and BEZ235 on MCF7 cells in response to TAM (10 nM) and IGF-1 (100 ng/mL) as indicated. (b) Inhibitory effects of kinase inhibitor BEZ35 at a dose range on cell proliferation of MCF7 cells in response to TAM (10 nM) and IGF-1 (100 ng/mL) as indicated. Original data are representative of three independent experiments. Data are expressed as means ± SD. [file bcr2883-S4.JPEG]
