# Supplementary material for: Elevated insulin-like growth factor 1 receptor signaling induces antiestrogen resistance through the MAPK/ERK and PI3K/Akt signaling routes
Source: Breast Cancer Res. 2011 May 19;13(3):R52. doi: 10.1186/bcr2883 (PMC3218939; doi:10.1186/bcr2883)
Supplement: Additional file 3 — Table S1. Effects of various kinase inhibitors and siRNA on IGF/E2/TAM-induced cell proliferation of MCF7/IGF-1R cells. [file bcr2883-S3.DOC]

| **kinase inhibitor** | **inhibited kinase** | **SRB (OD 510nm)** | **p value** | **fold change** |
| --- | --- | --- | --- | --- |
| (-) |  | 3.061 |  | 1.000 |
| BMS-536024 | IGF-1R | 0.752 | 0.0002 | -3.741 |
| U0126 | MEK | 1.391 | 0.0014 | -2.200 |
| PD 98059 | MEK | 1.793 | 0.0011 | -1.707 |
| AZD-6244 | MEK | 1.450 | 0.0002 | -2.111 |
| PD-184352 | MEK | 0.934 | 0.0014 | -3.276 |
| BEZ235 | PI3K | 0.860 | 0.0005 | -3.559 |
| PI 103 | PI3K | 0.762 | 0.0002 | -4.015 |
|  | | | | |
| **siRNA (SMARTpool)** | **targeted protein/kinase** | **SRB (OD 510nm)** | **p value** | **fold change** |
| siCtrl |  | 2.544 |  | 1.000 |
| *IGF1R* | IGF-1R | 1.400 | 0.0179 | -1.817 |
| *IRS1* | IRS-1 | 1.581 | 0.0315 | -1.609 |
| *MAP3K5* | ASK1 | 3.491 | 0.0299 | 1.372 |
| *PIK3R1* | PIK reg class IA | 3.345 | 0.0263 | 1.315 |
| *GSK3B* | GSK3B | 3.789 | 0.0373 | 1.489 |
| *PIK3CA* | PI3K cat class A | 0.698 | 0.0009 | -3.643 |
| *PTEN* | PTEN | 3.568 | 0.0398 | 1.402 |
| *AKT1* | AKT | 1.596 | 0.0410 | -1.594 |
| *PDPK1* | PDK | 1.142 | 0.0045 | -2.406 |
| *RPS6KA2* | p90Rsk | 1.403 | 0.0136 | -1.813 |
| *CHUK* | IKK-alpha (cat) | 3.354 | 0.0396 | 1.318 |
| *FRAP1* | mTOR | 1.368 | 0.0101 | -1.860 |
| *RPS6KB1* | p70 S6 kinase1 | 1.562 | 0.0448 | -1.629 |
| *Raf1* | c-Raf | 1.579 | 0.0450 | -1.611 |
| *MAPK1* | ERK2 | 1.469 | 0.0222 | -1.731 |

MCF7/IGF1R cells were treated with TAM (1 μM), E2 (1 nM) and IGF-1 (100 ng/ml). Cell proliferation was measured with the SRB assay. siCtrl, non-targeting siRNA control.
